# Experimental data of thermal cracking of soybean oil and blends with hydrogenated fat

**DOI:** 10.1016/j.dib.2018.01.054

**Published:** 2018-01-31

**Authors:** R.F. Beims, V. Botton, L. Ender, D.R. Scharf, E.L. Simionatto, H.F. Meier, V.R. Wiggers

**Affiliations:** a Chemical Engineering Department, University of Blumenau, Blumenau, SC, Brazil; b Chemistry Department, University of Blumenau, Blumenau, SC, Brazil

## Abstract

This article presents the experimental data on the thermal cracking of soybean oil and blends with hydrogenated fat. Thermal cracking experiments were carried out in a plug flow reactor with pure soybean oil and two blends with hydrogenated fat to reduce the degree of unsaturation of the feedstock. The same operational conditions was considered. The data obtained showed a total aromatics content reduction by 14% with the lowest degree of unsaturation feedstock. Other physicochemical data is presented, such as iodine index, acid index, density, kinematic viscosity. A distillation curve was carried out and compared with the curve from a petroleum sample.

**Specifications Table**

**Table:** 

Subject area	*Alternative fuels*
More specific subject area	*Thermal cracking (pyrolysis) of triglycerides*
Type of data	*Figures and tables*
How data was acquired	*Experiments and physicochemical analysis*
Data format	*Raw and tabulated data collection*
Experimental factors	*Yield of thermal cracking fractions (coke, bio-oil and bio-gas) and physicochemical properties of the products*
Experimental features	*Thermal cracking of triglycerides with different degrees of unsaturation (soybean oil and blends of soybean oil and hydrogenated fat)*
Data source location	
Data accessibility	*Data is with this article*
Related research article	*Beims et al., Effect of degree of triglyceride unsaturation on aromatics content in bio-oil*[1].

**Value of the data**

• This data provides a comparison between the thermal cracking of triglycerides with different degrees of unsaturation.
• Information regarding aromatics content due to different degrees of unsaturation in the triglycerides.
• The data presented details physicochemical properties of the bio-oil generated.

## 1 Data

**Fig. 1:**
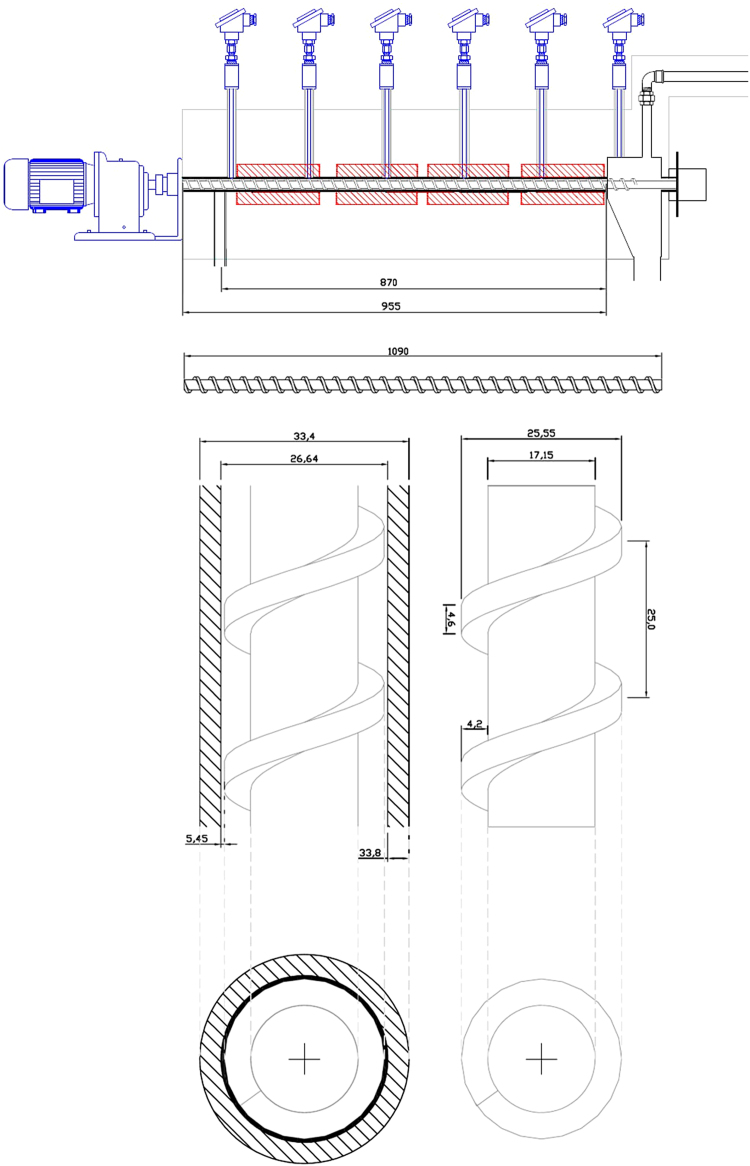
Reactor dimensions.

Fig. 1 illustrates the main dimensions of the thermal cracking reactor. Table 1 presents the yields of bio-oil, bio-gas and coke, as well as the operational conditions of each experiment. Table 2 shows the physicochemical properties of the bio-oils produced. Table 3 describes the fatty acids distribution in soybean oil. Table 4 has the oxygen content of bio-oil and bio-gas produced. Gas chromatography methods employed are described in Table 5. The bio-gas composition of each sample (Table 6) and the compounds distribution by carbon number (Table 7) were measured. The aromatic content in bio-oil is presented in Table 8 and the carboxylic acids in Table 9. Table 10 presents the distillation curve as well as the properties estimated through its data.

**Table 1:** Thermal cracking experiments.

Exp. number	Sample	Temperature (°C)	Mass flow (g/h)	Yield (%)		X_C_ (%)
				X_L_ (%)	X_G_ (%)	
1	SO 100	524.82 ± 0.07	316.29	62.97	22.49	14.54
2	SO 100	524.55 ± 0.36	322.66	61.24	26.40	12.37
3	SO 100	524.50 ± 0.63	335.22	59.68	22.24	18.08
4	SH 90:10	524.81 ± 0.11	300.00	59.47	22.99	17.54
5	SH 90:10	524.71 ± 0.20	322.58	64.84	22.43	12.74
6	SH 90:10	524,87 ± 0.13	327.18	67.99	20.80	11.21
7	SH 80:20	524,75 ± 0.19	315.79	69.56	20.00	10.44
8	SH 80:20	524.83 ± 0.19	320.66	66.39	23.41	10.20
9	SH 80:20	524.88 ± 0.32	333.17	67.52	24.05	8.43

**Table 2:** Physicochemical properties of the bio-oils produced.

Exp. number	Sample	II (gI_2_/100 g sample)	AI (gKOH/100 g sample)	ρ (kg/m^3^)	ν (mm^2^/s)
1	SO 100	176.28	211.12	0.846	24.614
2	SO 100	181.34	209.87	0.823	25.502
3	SO 100	179.34	211.89	0.842	24.743
4	SH 90:10	132.84	195.64	0.869	28.088
5	SH 90:10	135.9	198.12	0.866	26.827
6	SH 90:10	132.12	194.13	0.854	28.101
7	SH 80:20	122.54	136.37	0.928	40.105
8	SH 80:20	118.9	133.9	0.885	35.062
9	SH 80:20	120.1	140.9	0.907	47.941

**Table 3:** Fatty acids distribution in soybean oil.

Fatty acid	MM (g/mol)	%	Number of atoms		
			C	H	O
Palmitic (C16:0)	256	4.40	16	32	2
Stearic (C18:0)	284	4.15	18	36	2
Oleic (C18:1)	282	27.15	18	34	2
Linoleic (C18:2)	280	47.60	18	32	2
Linoleic (C18:3)	278	6.70	18	30	2
Average MM	272.12				
Triglyceride MM	870.64

**Table 4:** Oxygen content of bio-oil and bio-gas produced.

Exp. number	Sample	Oxygen content (wt%)	
		Bio-gas	Bio-oil
1	SO 100	0.033	16.12
2	SO 100	0.012	17.52
3	SO 100	0.015	17.85
4	SH 90:10	0.147	12.81
5	SH 90:10	0.175	10.89
6	SH 90:10	0.087	13.50
7	SH 80:20	0.058	14.52
8	SH 80:20	0.075	13.63
9	SH 80:20	0.083	13.42

**Table 5:** Gas chromatography methods.

Sample	Column	Carrier gas	Oven heating method	Injector temperature (°C)	Detector temperature (°C)
Bio-gas	Carboxen 1000 column (5 m × 2 mm, stainless steel)	Argon	Initially 40.0 °C (for 6 min), increasing to 220 °C at a ramp of 20 °C min^−1^. Temperature was kept at 220 °C for 20 min.	100	200
Bio-oil	RTX-1 capillary column (30 m × 0.32 mm × 3.00 ηm)	Helium	Initially 50 °C (for 2 min), increasing to 280 °C at a ramp of 5 °C min^−1^. Temperature was kept at 220 °C for 20 min.	100	200
Bio-oil (carboxylic acids)	RTX-5 capillary column (30 m × 0.25 mm × 3.00 ηm)	Helium	Initially 50 °C (for 2 min), increasing to 280 °C at a ramp of 5 °C min^−1^. Temperature was kept at 280 °C for 12 min.	250	290

**Table 6:** Bio-gas composition.

Exp. number	Sample	CO (vol%)	CH_4_ (vol%)	CO_2_ (vol%)	H_2_ (vol%)	C_2_–C_4_ (vol%)
1	SO 100	3.601	1.73	1.7391	4.442	88.4879
2	SO 100	1.082	0.425	0.8379	0	97.6551
3	SO 100	1.651	0.859	0.7975	3.98	92.7125
4	SH 90:10	16.74	7.919	7.106	6.645	61.59
5	SH 90:10	10.608	5.525	3.7266	5.746	74.3944
6	SH 90:10	20.849	10.509	7.7699	7.212	53.6601
7	SH 80:20	9.724	5.256	3.8665	5.531	75.6225
8	SH 80:20	6.62	3.209	2.7865	4.717	82.6675
9	SH 80:20	9.088	4.453	3.1573	5.158	78.1437

**Table 7:** Compounds by carbon number.

Compounds by carbon number	SO 100 (vol%)	SH 90:10 (vol%)	SH 80:20 (vol%)
Below C8	32.997 ± 4.69	28.061 ± 1.93	30.733 ± 2.55
C8–C9	7.976 ± 0.98	6.766 ± 0.35	6.946 ± 1.66
C9–C10	9.423 ± 1.76	9.409 ± 0.60	8.263 ± 1.12
C10–C11	6.857 ± 1.04	7.352 ± 0.63	6.319 ± 0.70
C11–C12	6.991 ± 1.75	8.780 ± 0.98	7.079 ± 0.91
C12–C13	5.636 ± 2.00	5.234 ± 0.25	6.545 ± 1.25
C13–C14	2.487 ± 0.23	1.580 ± 0.43	1.042 ± 0.44
C14–C15	4.783 ± 0.53	6.451 ± 0.94	4.955 ± 0.73
C15–C16	4.528 ± 0.77	3.754 ± 1.12	6.342 ± 1.26
C16–C17	3.633 ± 0.65	3.617 ± 1.22	5.090 ± 1.04
C17–C18	2.945 ± 0.42	4.483 ± 0.50	4.283 ± 0.23
C18–C19	2.055 ± 0.60	2.972 ± 0.52	1.963 ± 0.46
Above C19	9.770 ± 2.42	11.541 ± 1.21	10.441 ± 2.79

**Table 8:** Aromatics compounds in bio-oil.

Sample	Benzene (vol%)	Toluene (vol%)	Ethylbenzene (vol%)	m-p-xylene (vol%)	o-xylene (vol%)	Total (vol%)
SO 100	0.325 ± 0.031	0.431 ± 0.055	0.230 ± 0.020	0.108 ± 0.015	0.581 ± 0.068	1.6757 ± 0.037
SH 90:10	0.251 ± 0.019	0.324 ± 0.006	0.188 ± 0.007	0.087 ± 0.006	0.638 ± 0.055	1.4876 ± 0.018
SH 80:20	0.250 ± 0.020	0.320 ± 0.070	0.170 ± 0.010	0.090 ± 0.012	0.600 ± 0.080	1.4300 ± 0.038

**Table 9:** Carboxylic acids in bio-oil.

Exp. number	Sample	Palmitic acid (vol.%)	Oleic acid (vol.%)	Stearic acid (vol.%)
1	OS 100	29.15	53.11	17.44
2	OS 100	28.09	56.64	15.27
3	OS 100	28.11	58.27	13.62
4	SH 90:10	24.03	46.25	29.72
5	SH 90:10	25.81	46.54	27.65
6	SH 90:10	23.52	51.76	24.72
7	SH 80:20	22.58	44.58	32.84
8	SH 80:20	22.08	44.82	33.1
9	SH 80:20	24.34	41.9	33.76

**Table 10:** Distillation curves data and properties estimated.

Distilled volume (%)	Sample			Petroleum
	SO 100	SH 90:10	SH 80:20	
	Temperature (°C)			
0	40.8	28.4	29.4	24.3
5	87.1	90.8	80.9	67
10	128.4	141.6	132.8	136.1
15	–	–	139.1	159.8
20	173.7	201.1	191.1	183.2
25	204	225.5	218	209.3
30	222.7	244.4	236.2	241
35	239.5	265.6	255.7	267.2
40	258.3	293.5	277.3	289.8
45	276.7	320.2	303.5	297.6
50	297.7	337.8	324.2	303.4
55	319.3	345.7	336.2	340.2
60	333.2	348.1	341.6	367.9
65	338.9	–	343	389.4
70	341.90	351.94	347.7	421.5
73	–	353.1	–	–
75	344.9	–	–	444.8
76	–	401.2	–	–
80	405.7	–	369.2	448.7
83	418.3	–	–	–
86	–	–	385.2	–
90	444.68	473.5913	445.9104	–
°API	37,5	32,4	24,6	25.9
VABP	287.2	309.9	297.3	327.8
MM	186.1	190.2	181.8	198.4

## 2 Experimental design, materials, and methods

### 2.1 Materials

Experiments were carried out with soybean oil and blends of commercial soybean oil with hydrogenated fat. The sample called SO 100 is composed entirely by soybean oil, while SH 90:10 and SH 80:20 are blends of soybean oil with hydrogenated fat (derived from soybean oil). The first blend has 10% (weight) of hydrogenated fat and the latter has 20% (weight).

### 2.2 Methods

All samples were fed in the reactor at 90 °C. Once hydrogenated fat is solid at room temperature, the preheat was necessary to maintain in the liquid state and avoid clogging in the reactor.

### 2.3 Thermal cracking reactor

Thermal cracking experiments were performed in a plug flow reactor, under isothermal and steady-state conditions (Table 1). It was considered similar operational conditions to all samples (constant feed mass flow and reactor temperature). Reactor main dimensions are shown in Fig. 1. Further details of the reactor were described by [2], [3].

The residence time (*t_res_*) is a relation between the reactor volume (*V_reactor_*) and volumetric flow (*q*):

(1) $$t_{\mathrm{res}}=\frac{V_{\mathrm{reactor}}}{q}$$

Volumetric flow can be written as,

(2) $$q=\frac{\dot{m}}{\overset{-}{\rho }}$$

where $\overset{-}{\rho }$ is derived from the Ideal Gas Law,

(3) $$\overset{-}{\rho }=\frac{P\overset{-}{\mathrm{MM}}}{\mathrm{RT}}$$

Thus,

(4) $$t_{\mathrm{res}}=\frac{V_{\mathrm{reactor}}}{\left(\frac{\dot{m}}{\rho }\right)}$$

where *P* is the reactor pressure, *R* is the universal gas constant, *T* is the reactor temperature, $\overset{-}{\mathrm{MM}}$ is the average molecular mass and *V_reactor_* is the reactor volume (considered as the region heated by the heating elements and where the thermal cracking occurs = 2.91E−4 m^3^).

The average molecular mass was estimated from the fractions of bio-oil and bio-gas and their average molecular mass, which was based on information on their composition obtained from gas chromatography analysis (Section 2.6).

(5) $$\overset{-}{\mathrm{MM}}=X_{\mathrm{BO}}{\overset{-}{\mathrm{MM}}}_{\mathrm{BO}}+X_{\mathrm{BG}}{\overset{-}{\mathrm{MM}}}_{\mathrm{BG}}$$

The average molecular mass of the bio-oil was estimated considering its composition in terms of the fractions and the average molecular mass of the compounds according to the number of carbon atoms in the chain. Each fraction was determined via a comparison with an n-alkane standard sample using GC-FID analysis:

(6) $${\overset{-}{\mathrm{MM}}}_{\mathrm{BO}}=\;\sum \;X_{i\_\mathrm{liq}}{\mathrm{MM}}_{i\_\mathrm{liq}}$$

The average molecular mass for the bio-gas was determined similarly to that of the bio-oil, where the fractions of the bio-gas compounds were determined by GC-FID/TCD.

(7) $${\overset{-}{\mathrm{MM}}}_{\mathrm{BG}}=\;\sum \;X_{i\_\mathrm{gas}}{\mathrm{MM}}_{i\_\mathrm{gas}}$$

where, ${\overset{-}{\mathrm{MM}}}_{\mathrm{BO}}$ is the average molecular mass of the bio-oil, ${\overset{-}{\mathrm{MM}}}_{\mathrm{i\_liq}}$ is the average molecular mass of each fraction in the liquid, $X_{\mathrm{i\_liq}}$ is the fraction of each compound in the liquid (separated by the number of carbon atoms in the chain), ${\overset{-}{\mathrm{MM}}}_{\mathrm{BG}}$ is the average molecular mass of the bio-gas, ${\overset{-}{\mathrm{MM}}}_{\mathrm{i\_gas}}$ is the average molecular mass of each fraction in the gas, $X_{\mathrm{i\_gas}}$ is the fraction of each compound in the gas.

### 2.4 Physicochemical properties

Several analyses were performed to determinate physicochemical properties of the bio-oil produced. EN14111 standard was considered for the iodine index (II) determination, which is associated to unsaturated compounds. A higher II suggests a higher number of double bounds in the molecular chain.

Acidity index (AI) values were obtained according to the method available in ASTM D 974/2008. *AI* is related to the presence of organic acids in bio-oil.

The specific gravity (*ρ*) of the bio-oil samples was evaluated according to ASTM D5355-95 (2012).

The kinematic viscosity (*ν*) of the bio-oil samples was determined using a Ford Viscosity Cup (n. 4), according to ASTM D1200.

### 2.5 Oxygen content in the bio-oil

The mass of oxygen in the bio-oil produced was estimated by mass balance, according to Eq. (8).

(8) $$m_{\mathrm{bio}-\mathrm{oil}}=m_{\mathrm{Oxygen}\_\mathrm{TG}}-m_{\mathrm{Oxygen}\_\mathrm{BG}}$$

where $m_{\mathrm{bio}-\mathrm{oil}}$, $m_{\mathrm{Oxygen\_TG}}$ and $m_{\mathrm{Oxygen\_BG}}$ are, respectively, the mass of oxygen in the bio-oil, triglyceride (biomass) and bio-gas. It was assumed that coke does not contain a significant amount of oxygen.

**Fig. 2:**
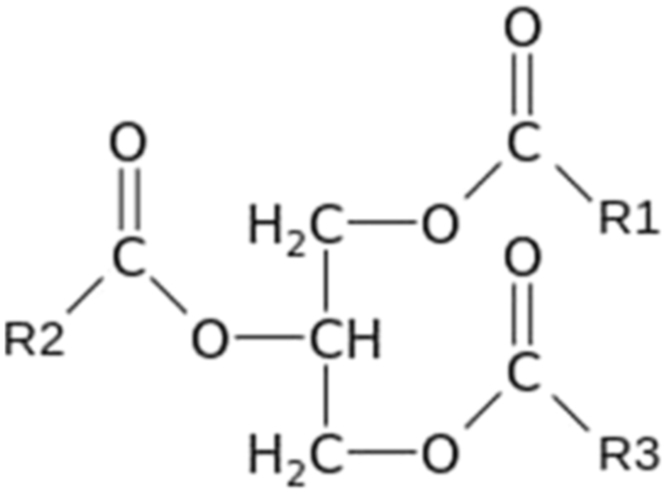
Triglyceride molecule.

The oxygen mass in the feedstock biomass in each experiment was estimated using Eqs. (9) and (10), considering that a triglyceride molecule has the format presented in Fig. 2, where R1, R2 and R3 are three different radicals (fatty acids), which have the distribution shown in Table 3.

(9) $$X_{\mathrm{oxygen}\_\mathrm{TG}}=\frac{{\mathrm{MM}}_{\mathrm{Oxygen}\_\mathrm{TG}}}{{\mathrm{MM}}_{\mathrm{TG}}}$$

and

(10) $$m_{\mathrm{oxygen}\_\mathrm{TG}}=m_{\mathrm{biomass}}X_{\mathrm{oxygen}\_\mathrm{TG}}$$

where ${\mathrm{MM}}_{\mathrm{Oxygen\_TG}}$ is the amount of oxygen in the triglyceride molecule [g/mol], ${\mathrm{MM}}_{\mathrm{TG}}$ is the molecular mass of triglyceride [g/mol], $X_{\mathrm{oxygen\_TG}}$ is the fraction of oxygen in the triglyceride [g_oxygen_/g_triglyceride_], $m_{\mathrm{biomass}}$ is the amount of biomass fed in the experiment [g_triglyceride_] and $m_{\mathrm{oxyge}n_{\mathrm{TG}}}$ is the amount of oxygen in the biomass [g_oxygen_].

Oxygen was present in the bio-gas as CO and CO_2_, both fractions being determined by GC-FID/TCD (Section 2.6). With the amount of bio-gas produced and its composition, the oxygen content can be determined by (11), (12), (13).

(11) $$X_{\mathrm{oxygen}\_\mathrm{CO}}=\frac{{\mathrm{MM}}_{\mathrm{Oxygen}\_\mathrm{CO}}}{{\mathrm{MM}}_{\mathrm{CO}}}$$

(12) $$X_{\mathrm{oxygen}\_\mathrm{CO}2}=\frac{{\mathrm{MM}}_{\mathrm{Oxygen}\_\mathrm{CO}2}}{{\mathrm{MM}}_{\mathrm{CO}2}}$$

(13) $$m_{\mathrm{oxygen}\_\mathrm{BG}}=\left(X_{o\mathrm{xygen}\_\mathrm{CO}}m_{\mathrm{CO}}\right)+\left(X_{\mathrm{oxygen}\_\mathrm{CO}2}m_{\mathrm{CO}2}\right)$$

where $m_{\mathrm{CO}}$ is the mass of CO in bio-gas [g], ${\mathrm{MM}}_{\mathrm{CO}}$ is the molecular mass of CO [g/mol], $m_{\mathrm{CO}2}$ is the mass of CO_2_ in bio-gas [g], ${\mathrm{MM}}_{\mathrm{CO}2}$ is the molecular mass of CO_2_ [g/mol], $X_{\mathrm{O\_CO}}$ is the fraction of oxygen in CO [g_oxygen_/g_CO_], $X_{\mathrm{O\_CO}2}$ is the fraction of oxygen in CO_2_ [g_oxygen_/g_CO2_] and ${\mathrm{MM}}_{\mathrm{Oxygen}}$ is the molecular mass of oxygen [g/mol].

### 2.6 Gas chromatography analyses

A Shimadzu® model GCMS-QP2010 Plus was employed to Gas chromatography analyses. Bio-gas samples were analyzed by gas chromatography-flame ionization detection/thermal conductivity detection (GC-FID/TCD), aiming at the identification of CO, CO_2_, methane, hydrogen and light hydrocarbons (C_2_–C_4_ range). Bio-oil samples were submitted to GC-FID to determine the compounds according to their carbon number distribution by comparison with n-alkane standards. The headspace technique was performed to identify the aromatic compounds. Lastly, carboxylic acids in the bio-oil were determined by GC–MS. Methods employed to all samples are described in Table 5.

### 2.7 Distillation curves

The distillation curve is an important tool to predict bio-oil properties aiming on its co-processing in a standard petroleum refinery [4]. Experiments were performed in an automatic vacuum distiller (B/R Instrument, model M690), based on the standards for petroleum characterization (ASTM D86-04b/2004, and AST D1160-02a, 2002). Minor modifications were considered: distillation rate (1 mL/min, instead of 4–5 mL/min) and volume of sample (200 mL, instead of 100 mL). The distillation curve of a petroleum sample originating from the Baúna's field (Brazil) was carried out for comparison purposes.

API gravity (an approach to measuring the specific gravity and it is also widely employed in the characterization of petroleum). The API gravity of crude oil and bio-oil samples was calculated using Eq. (14).

(14) $$\mathrm{API}=\frac{141.5}{\mathrm{SG}}-131.5$$

where *SG* is the specific gravity (or density) at 20/4 °C.

Volumetric average boiling point (VABP) is an intermediate boiling point of a crude oil). *VABP* is determined using Eq. (15).

(15) $$\mathrm{VABP}=\frac{T_{10}+T_{30}+T_{50}+T_{70}+T_{90}}{5}$$

where, *T*_10_, *T*_30_, *T*_50_, *T*_70_, *T*_90_ are the temperatures for each distillated volume of the sample (subscript number %).

The mean average boiling point (*MeABP*), which allows the estimation of the molecular mass of the oil sample (*MM*). Given that the *VABP* is calculated from the distillation curve data (Eq. 15), the *MeABP* is calculated as:

(16) MeABP=VABP−∆

where

(17) $$\ln∆=-0.94402-{0.00865(\mathrm{VABP}-32)}^{0.6667}+{\left(2.99791\left(\frac{T_{90}-T_{10}}{90-10}\right)\right)}^{0.333}$$

The molecular mass of a crude oil sample can be calculated using the Pedersen correlation [5], given by Eq. (18):

(18) $$\mathrm{MM}=42.965\left[e^{2.097\times 10^{-4}\left(\mathrm{MeABP}\right)-7.78712\left(\mathrm{SG}\right)+2.08476\times 10^{-3}\left(\mathrm{MeABP}\right)\left(\mathrm{SG}\right)}\right]({\mathrm{MeABP})}^{1.26007}{(\mathrm{SG})}^{4.98308}$$
